# Static and Dynamic Functional Network Connectivity in Parkinson's Disease Patients With Postural Instability and Gait Disorder

**DOI:** 10.1111/cns.70115

**Published:** 2024-11-10

**Authors:** Bo Shen, Qun Yao, Yixuan Zhang, Yinyin Jiang, Yaxi Wang, Xu Jiang, Yang Zhao, Haiying Zhang, Shuangshuang Dong, Dongfeng Li, Yaning Chen, Yang Pan, Jun Yan, Feng Han, Shengrong Li, Qi Zhu, Daoqiang Zhang, Li Zhang, Yun‐cheng Wu

**Affiliations:** ^1^ Department of Geriatrics Affiliated Brain Hospital of Nanjing Medical University Nanjing China; ^2^ Key Laboratory of Brain‐Machine Intelligence Technology, Ministry of Education Nanjing University of Aeronautics and Astronautics Nanjing China; ^3^ Department of Neurology Shanghai General Hospital of Nanjing Medical University Shanghai China; ^4^ Department of Neurology Affiliated Brain Hospital of Nanjing Medical University Nanjing China; ^5^ Medical Basic Research Innovation Center for Cardiovascular and Cerebrovascular Diseases Ministry of Education China; ^6^ International Joint Laboratory for Drug Target of Critical Illnesses, School of Pharmacy Nanjing Medical University Nanjing China; ^7^ College of Computer Science and Technology Nanjing University of Aeronautics and Astronautics Nanjing China

## Abstract

**Aims:**

The exact cause of the parkinsonism gait remains uncertain. We first focus on understanding the underlying neurological reasons for these symptoms through the examination of both static functional network connectivity (SFNC) and dynamic functional network connectivity (DFNC).

**Methods:**

We recruited 64 postural instability and gait disorder‐dominated Parkinson's disease (PIGD‐PD) patients, 31 non‐PIGD‐PD (nPIGD‐PD) patients, and 54 healthy controls (HC) from Nanjing Brain Hospital. The GIFT software identified five distinct independent components: the basal ganglia (BG), cerebellum (CB), sensory networks (SMN), default mode network (DMN), and central executive network (CEN). We conducted a comparison between the SFNC and DFNC of the five networks and analyzed their correlations with postural instability and gait disorder (PIGD) symptoms.

**Results:**

Compared with nPIGD‐PD patients, the PIGD‐PD patients demonstrated reduced connectivity between CEN and DMN while spending less mean dwell time (MDT) in state 4. This is characterized by strong connections. Compared with HC, PIGD‐PD patients exhibited enhanced connectivity in the SFNC between CB and CEN, as well as the network between CB and DMN. Patients with PIGD‐PD spent more MDT in state 1, which is characterized by few connections, and less MDT in state 4. In state 3, there was an increase in the functional connectivity between the CB and DMN in patients with PIGD‐PD. The nPIGD patients showed increased SFNC connectivity between CB and DMN compared to HC. These patients spent more MDT in state 1 and less in state 4. The MDT and fractional windows of state 2 showed a positive link with PIGD scores.

**Conclusion:**

Patients with PIGD‐PD exhibit a higher likelihood of experiencing reduced brain connectivity and impaired information processing. The enhanced connection between the cerebellum and DMN networks is considered a type of dynamic compensation.

## Graphical abstract

We first compared the DFNC and SFNC of the three networks and examined their associations with tremor symptoms. PIGD‐PD patients were more likely to remain in the condition of sparse connection. The enhanced connection between the cerebellum and cognitive networks is considered a type of dynamic compensation.

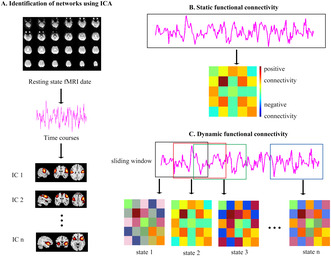

## 1 Introduction

The loss of dopamine‐producing cells in the substantia nigra, a specific region of the midbrain, results in Parkinson's disease (PD), a progressive neurodegenerative disorder that causes a gradual decline in motor function [1]. Nevertheless, PD also encompasses nonmotor symptoms such as cognitive decline, mood disorders, sleep disruptions, and autonomic dysfunction [2]. Parkinsonian gait is distinguished by reduced propulsive force, which leads to sluggish and diminutive steps, a stooping posture, inadequate foot clearance, and compromised coordination between limbs. However, the causes of the gait remain unexplained [3].

Research involving functional MRI (fMRI) has made substantial contributions to our understanding of the brain mechanisms for gait impairment in PD [4]. Specifically, people with PD exhibited reduced activity in the supplementary motor area while doing gait activities [5]. Furthermore, the cerebellum motor region exhibited the most reliable activation in relation to gait, suggesting a potential compensatory function in locomotion for individuals with PD [6]. One study examined the neurological, cognitive, and functional brain activity associated with dual‐task impairments in patients with gait disorders. Findings from this study indicate that these patients had impaired gait ability, especially when turning, while also demonstrating heightened activation of the inferior frontal gyrus and supplementary motor region. The finding indicated that enhanced recruitment of motor and cognitive networks is linked to improved gait performance in these patients [7]. However, the connection between the motor and cognition networks with regard to gait in PD has not yet been determined.

In most previous studies, it was assumed that functional connections remained unchanged throughout fMRI scanning. Static Functional Network Connectivity (SFNC) is the measurement of the temporal correlation between distant neurophysiological events, often calculated as a single average over a specific time period [8]. It is commonly employed to characterize the consistent, fundamental levels of synchronization among various parts of the brain. Dynamic Functional Network connection (DFNC) is an expansion of this idea that focuses on investigating alterations in functional connections over a period of time. This approach can uncover temporary, repetitive patterns of synchronization among brain regions that may be associated with various cognitive states or processes [9]. A first study conducted in 2017 demonstrated that patients with PD display dynamic connectivity patterns in comparison to individuals without the condition. Patients with PD exhibit reduced mean dwell time (MDT) of hypo‐connectivity and demonstrate heightened transitions between various connectivity states [10]. This indicates a more fluctuating and less consistent pattern of brain connection, which may be the underlying cause of the motor impairments reported in these patients. Furthermore, a study discovered a direct correlation between the fluctuating connectivity of the Default Mode Network (DMN) and the cognitive ability of visuospatial memory in patients with PD [11]. These findings indicate that alterations in brain dynamics during periods of inactivity may be directly associated with cognitive abilities in individuals with PD. The study suggested that the measurement of dynamic brain activity using DFNC could serve as a neural indicator of motor and cognitive skills in individuals with PD [12].

An investigation into both static and dynamic functional connectivity could offer valuable insights into the fundamental brain causes of symptoms, such as problems with gait. As far as we are aware, this study is the first to examine the relationship between these networks and their role in patients with gait problems.

## 2 Methods

### 2.1 Participants

Between the beginning of 2015 and the middle of 2019, a study was conducted at the geriatric department of the Brain Hospital affiliated with Nanjing Medical University, recruiting 95 persons diagnosed with PD and 54 healthy controls (HC) for control comparison. All participants were verified to be right‐handed and provided written consent after being fully told about the nature of the study. This study was approved by the medical research ethical committee of Nanjing Brain Hospital, Nanjing, China. According to the MDS established criteria, the study only included patients with a confirmed diagnosis of idiopathic PD [13]. Exclusion criteria were the presence of significant cognitive deficits and other neurological disorders, severe mental illness, including anxiety and depression, or failure to fulfill the safety criteria for MRI scanning.

### 2.2 Neuropsychological and Neuropsychiatric Assessment

Participants underwent a specific neuropsychological assessment to evaluate motor symptoms using the Unified Parkinson's Disease Rating Scale (UPDRS) [14]. This evaluation was performed in two conditions: one when the participant was on medicine and another where they were off medication. In the off condition, the participant had to stop taking anti‐PD medications for at least 24 h and extended‐release formulations for 72 h. Patients with a mean tremor score to the mean postural instability and gait disorder (PIGD) score ratio of less than 1 are characterized as PIGD‐dominant PD(PIGD‐PD). In contrast, others are recognized as possessing the non‐PIGD PD (nPIGD‐PD) subtype [15]. The study included 64 PIGD‐PD, 31 nPIGD‐PD patients. In addition, we assessed the cognitive abilities of all individuals by administering both the Mini‐Mental State Examination (MMSE) and the Montreal Cognitive Assessment (MoCA) [16].

### 2.3 Data Acquisition and Preprocessing

In our refined study approach, we captured imaging data on a 3.0T Siemens Verio MRI system equipped with an eight‐channel head coil. These images were taken during the patients' on‐medication phase to reduce potential movement artifacts. The imaging protocol utilized a turbo field echo sequence with specific imaging parameters, which included a repetition time of 1900 ms and an echo time of 2.48 ms. The field of view was set at 250 × 250 mm with a matrix resolution of 256 × 256, capturing 176 slices in total without any gaps between them, resulting in 176 total image volumes. We utilized an echo‐planar imaging sequence with a repetition time of 2000 ms and an echo time of 30 ms for the resting‐state fMRI scans. The scans had a field of view of 240 × 240 mm and a matrix size of 64 × 64 mm, without any gaps. Each participant's scan consisted of 240 volumes and had a total duration of 8 min and 6 s. Participants were given explicit instructions to maintain closed eyes, refrain from engaging in active cognitive processes, and prevent themselves from falling asleep during the scan. Noise‐canceling headphones were employed, and the range of head motion was restricted with the assistance of foam padding.

The DPARSF software, which is executed on MATLAB R2013b, was utilized to perform the data preprocessing [17]. The preprocessing process had several steps, such as getting rid of the starting time points, fixing the timing of the slices, estimating the head's movement, normalizing the space using a certain alignment method and a 3 mm × 3 mm voxel size, and smoothing the space using an 8 mm full‐width half‐maximum Gaussian filter. In addition, we conducted detrending to eliminate linear trends and implemented a frequency filter with a pass band ranging from 0.01 to 0.08 Hz. This process involved regression analysis on signals from white matter and cerebrospinal fluid, as well as on 24 parameters related to head motion. It is worth mentioning that none of the participants showed any head movement greater than 3 mm in any direction or 3° of rotation.

### 2.4 Identification of Intrinsic Connectivity Networks

After preprocessing the data, the resting‐state fMRI (rs‐fMRI) of all participants was analyzed using spatial independent component analysis (ICA) as implemented in the GIFT software [18]. This analysis decomposed the data into functional networks, each with a unique time course profile. Principal component analysis encompasses two distinct procedures for data reduction: subject‐specific and group‐level phases [19]. The subject‐specific data were combined over time after being reduced to 120 components. In addition, 100 independent components (ICs) were identified at the group level using the expectation–maximization approach implemented in GIFT. The method was conducted 100 times to assess the dependability and consistency of the infomax principal components analysis algorithm in ICASSO. The resulting components were grouped together to assess their reliability, and only components with values exceeding 0.80 were selected. Subject‐specific spatial maps and time sequences were generated using the back‐reconstruction technique. By employing a preestablished method, we identified significant intrinsic connection networks inside the 100 ICs. Initially, we conducted a manual examination to ensure that the peak activation coordinates were primarily situated in gray matter and did not significantly overlap with vascular, ventricular, or edge regions associated with artifacts. Subsequently, if the time course was predominantly influenced by low‐frequency fluctuations, specifically with a power ratio of 0.10 Hz to 0.15–0.25 Hz, we categorized the selected independent components into five functional networks: basal ganglia (BG), cerebellum (CB), sensorimotor network (SMN), DMN, and central executive network (CEN).

### 2.5 SFNC Analysis of Two Groups

The MANCOVAN toolbox in the GIFT software was used for the SFNC analysis to look at differences in functional connectivity within the predefined spatial ICs. Detrending, despiking, and applying a low‐pass filter with a frequency range of 0.01–0.15 Hz were the initial processing steps for specific ICs' time courses. Afterward, a two‐sample t‐test was used to evaluate the disparity between PIGD‐PD patients, nPIGD‐PD patients, and HC (*p* < 0.05, false discovery rate [FDR] correction).

### 2.6 DFNC Analysis of Two Groups

The dynamic functional connectivity investigation was analyzed using a sliding window approach and k‐means clustering. Consistent with previous studies [20], the resting state data were divided into segments of 22 repetition times (44 s) each, with a one repetition time increment. This segment length has been demonstrated to strike a favorable balance between the accuracy of the correlation matrix estimation and the ability to detect changes over time. Furthermore, we incorporated an extra L1 norm into the precision matrix to encourage sparsity inside the 100‐repetition graphic LASSO framework. In order to standardize the variability before conducting further research, all functional connectivity matrices were converted into z scores using Fisher's z transformation after calculating DFNC. We looked at the temporal properties of DFNC states by finding the average length of time spent in each state, the share of time spent in each state, and the number of transitions (NT) between states. The MDT is the count of consecutive windows that pertain to a single state. The fractional windows represent the count of total windows that belong to a specific state. NT refers to the number of transitions between states, which indicates the dependability of each state. Statistical analysis was performed using a two‐sample *t*‐test with FDR correction (*p* < 0.05) to decipher the differences among the three groups: HC, PIGD‐PD, and nPIGD‐PD. We specifically observed the variations in MDT, fractional windows, and NT while matching the groups for age, gender, and education.

### 2.7 Clinical and Neuropsychological Data Analysis

The statistical analyses were performed using SPSS Statistics, version 20.0 (Chicago, IL, USA). ANOVA analysis helped compare the age difference between patients with the PIGD subtype, nPIGD subtype, and HC. The chi‐squared test helped provide the gender differences. The Mann–Whitney test helped compare the differences in tremor PIGD scores. The two‐sample *t*‐tests compared the differences between the two subtypes in terms of the duration, UPDRS scores, and H&Y stage. We conducted Pearson's correlation analyses between altered connection features and clinical symptoms in patients with PIGD‐PD. A statistical significance criterion was created, with a threshold of *p* < 0.05.

## 3 Results

### 3.1 Demographic, Clinical, and Movement Characteristics

The study sample size included 64 PIGD‐PD patients, 31 nPIGD‐PD patients, and 54 HC. No major differences could be detected between HC, PIGD‐PD, and nPIGD‐PD patients regarding age, education, or gender. Similarly, no distinctions could be observed between the MMSE and MOCA scores between the two subgroups. The PIGD subgroup had more tremor scores than the nPIGD subgroup. Data collection occurred during the individuals' medication period, with the dates in detail provided in Table 1.

**TABLE 1:** Demographic and neuropsychological characteristics of all subjects.

Group	PIGD‐PD (*n* = 64)	nPIGD‐PD (*n* = 31)	HC (*n* = 54)	*p*
Age (year)	60.13 ± 9.20	62.13 ± 6.20	59.51 ± 6.15	0.31^a^
Sex (male/female)	30/34	11/20	26/28	0.49^b^
Education, year	10.72 ± 3.72	9.22 ± 4.50	10.17 ± 3.60	0.21^a^
MMSE	27.81 ± 2.11	27.20 ± 1.74	27.33 ± 1.75	0.24^a^
MoCA	25.39 ± 2.79	24.71 ± 2.34	24.52 ± 2.73	0.19^a^
Duration (year)	8.19 ± 5.42	6.90 ± 3.79		0.24^c^
UPDRS	56.80 ± 20.56	50.68 ± 16.39		0.15^c^
Hoehn and Yahr	2.80 ± 0.79	2.63 ± 0.97		0.35^c^
Tremor scores	6 (0,12)	9 (3,18)		< 0.01
PIGD scores	9 (3,16)	6 (2,13)		0.07

*Note: p* ^a^ value among three groups was obtained by ANOVA analysis, *p* ^b^ value for gender difference was obtained by chi‐squared test, *p* ^c^ value was obtained by two‐sample *t*‐test, others were obtained by Mann–Whitney test.

Abbreviations: MMSE, Mini‐Mental State Examination; MOCA, Montreal Cognitive Assessment; PIGD, postural instability and gait difficulty; UPDRS, unified Parkinson's disease rating scale.

### 3.2 Static Functional Connectivity State Analysis

**FIGURE 1:**
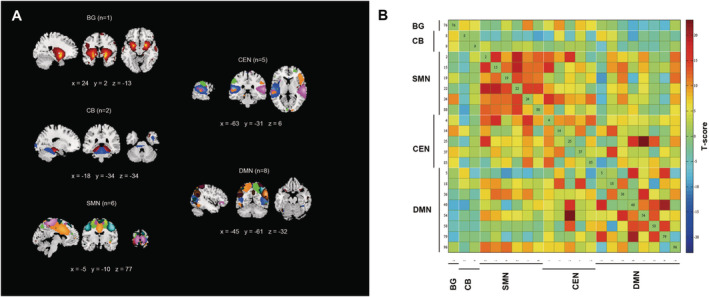
The 22 independent components identified by a group ICA. (A) Five functional networks (BG, basal ganglia; CB, cerebellar; CEN, central executive network; DMN, default mode network; SMN, sensorimotor network) were identified by grouping subsets of the 22 independent components. (B) Group averaged static functional connectivity between independent component pairs was computed using the entire resting state data. The index numbers of independent components are written on the left sides of the matrix, along with a color‐coded legend that matches the overlaid colors of the spatial maps in (A).

**FIGURE 2:**
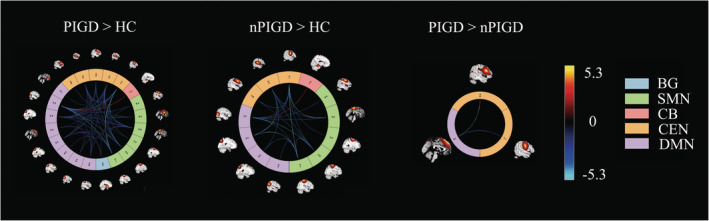
Group differences in SFNC strength between PD patients and HC. Each square color represents one of the five networks. BG, basal ganglia; CB, cerebellar; CEN, central executive network; DMN, default mode network; SMN, sensorimotor, the red line represents the increased functional connectivity, and the blue line represents the decreased functional connectivity.

Figure 1A illustrates the spatial maps of all 22 ICs that were determined using group ICA. The 22 ICs were divided into five networks: BG (IC 76), CB (IC 8, 9), SMN (IC 2, 15, 19, 22, 24, 80), CEN (IC 4, 14, 25, 37, 85), and DMN (IC 5, 18, 36, 40, 54, 58, 79, 96). Figure 1B illustrates the group average of the static functional connectivity network between ICs that was generated over the whole scan. Compared with nPIGD, the functional connectivity between CEN and DMN was decreased in the PIGD subgroup. In comparison to HC, patients with PIGD‐PD showed reduced connectivity within the SMN, DMN, and CEN. Additionally, there was decreased connection within the BG, SMN, DMN, and CEN networks in PIGD‐PD patients. We also saw better functional connectivity between the CB‐DMN and BG‐CEN networks, which was statistically significant. Compared with HC, the nPIGD subgroup possessed similar connectivity. However, there was decreased connectivity within and among SMN, DMN, and CEN networks. Additionally, the functional connectivity between CB and DMN networks was elevated (*p* < 0.05, FDR‐corrected) (Figure 2).

### 3.3 Dynamic Functional Connectivity State Analysis

**FIGURE 3:**
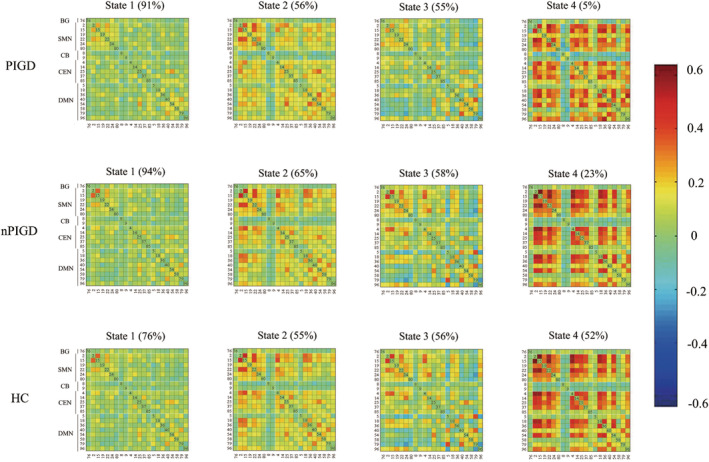
Functional connectivity state results of PIGD‐PD patients, nPIGD‐PD patients, and HC. Group‐specific cluster centroids matrices for each state. Each subject can enter one to four of the defined states throughout the entire scanning. The number of subjects for each group entering the specific state was presented below the matrices. The red color represents the positive correlation, whereas the blue color represents the negative correlation.

**FIGURE 4:**
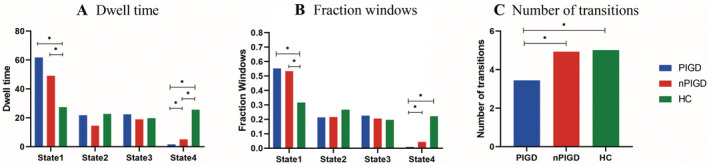
Temporal properties of functional connectivity state analysis. (A) The MDT of total subjects spent in each state. (B) The mean fractional windows of total subjects spent in each state are measured by percentage. (C) NT is depicted for the PIGD‐PD patients, nPIGD‐PD patients, and HC. Asterisks indicate a significant group difference (*p* < 0.05, FDR corrected).

**FIGURE 5:**
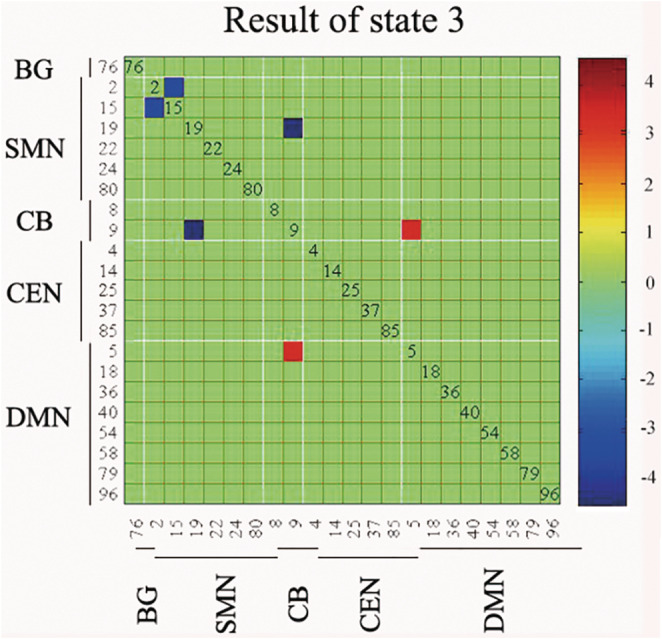
Dynamic functional connectivity difference between PIGD‐PD patients and HC. BG, basal ganglia; CB, cerebellar; CEN, central executive network, DMN, default mode network; SMN, sensorimotor, the red color represents the increased functional connectivity, and the blue line represents the decreased functional connectivity.

The optimal number of clusters, determined using the elbow criterion of the cluster validity index, was estimated to be four (*k* = 4). The cluster validity index measures the similarity of a point to other points within its own cluster compared to points in other clusters. The DFNC windows of each subject were categorized into one of four states based on their resemblance to the cluster centroids. State 1, which was the most frequent and least interconnected, and State 4, which was the least frequent and most interconnected, were included in the classification. The frequency and strength of the linkage between states 2 and 3 were similar, as shown in Figure 3. However, not all participants displayed all four states. We conducted a comparison of the differences in functional connection strength between the two groups in each state. The statistical significance threshold was set at *p* < 0.05, and the results were corrected with FDR. Compared with nPIGD‐PD, the PIGD‐PD showed reduced MDT and fraction windows in state 4. Moreover, the PIGD subgroup depicted decreased NT compared with nPIGD. PIGD‐PD and nPIGD‐PD patients demonstrated a prolonged MDT in state 1 and a reduced MDT in state 4. Compared to HC, fraction windows were increased and decreased in state 1 and state 4, respectively. The two groups also show notable disparity in NT as depicted in Figure 4. In state 3, only individuals with PIGD‐PD showed reduced connections within the SMN and CB‐SMN networks. Additionally, there was an increase in connectivity between the CB and DMN. There was no connectivity difference in the four states between nPIGD‐PD patients and HC. These findings were statistically significant (*p* < 0.05, FDR‐corrected) and are illustrated in Figure 5.

### 3.4 Correlation Analysis

**FIGURE 6:**
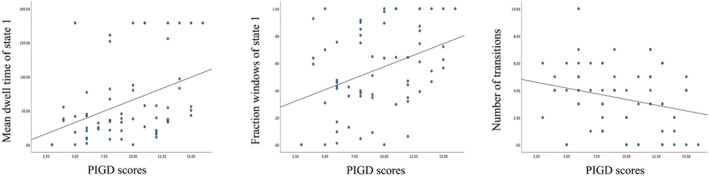
Correlation results between PIGD symptoms and temporal properties. The MDT and fraction windows of State 2 showed a positive correlation with the PIGD scores in individuals with PIGD‐PD patients. Conversely, the NT was negatively connected with the PIGD scores in these subjects (*p* < 0.05).

Further research on the relationships between dynamic functional connectivity metrics and the clinical features of the PIGD‐PD patients revealed a positive correlation between the PIGD score and the MDT and fractional windows of State 1. In addition, there was a negative correlation between the NT among states and the PIGD score. We found no correlation between dynamic network connectivity features and cognitive scores. This suggests a connection between alterations in dynamic states and the severity of motor symptoms in PIGD‐PD group (Figure 6).

## 4 Discussion

This study is the first attempt to integrate network analysis with state and dynamic connectivity in order to examine the changes in networks that are linked to gait problems. Patients with PIGD‐PD exhibited atypical SFNC and DFNC in both the inter‐ and intra‐networks. (1) PIGD‐PD patients showed reduced SFNC between CEN and DMN compared to nPIGD‐PD patients. Compared to HC, PIGP‐PD patients exhibited increased SFNC connectivity between CB and CEN, along with the network between CB and DMN. (2) Compared to HC, PIGD‐PD and nPIGD‐PD patients spent more dwell time in state 1, which was characterized by few connections. Moreover, there was less dwell time in state 4, which was characterized by strong connections. PIGD‐PD patients demonstrated lesser state 1 MDT and fraction windows between the two subgroups. (3) In state 3, there was an increase in the functional connectivity between the CB and DMN in patients with PIGD‐PD, whereas there was a decrease in the functional connectivity between the SMN and CB. (4) The MDT and fractional windows of state 1 showed a positive link with PIGD scores, whereas NT had a negative correlation.

Initially, we observed a reduction in functional connection between the CB and SMN networks during state 3 as well as a decrease in connectivity within the SMN networks. A previous study has repeatedly demonstrated a reduction in functional connectivity within the SMN, specifically in the left inferior parietal and primary somatosensory cortex [21]. In addition, PD patients exhibit reduced connections between the SMN and visual networks, these reductions in connection appear to be partially restored when patients are undergoing dopaminergic medication [22]. The alterations in both regional and distant neural pathways are believed to be linked to compromised sensory integration for motor function in PD [23]. Furthermore, studies on PD patients experiencing excessive daytime sleepiness have revealed changes in the way different brain regions communicate with each other, specifically involving the SMN network [24].

Decreased functional connectivity was observed between CEN and DMN networks. The CEN network is critical in executive functions, including goal‐directed behavior, impulsivity, and maintaining and manipulating working memory information [25]. The DMN network involves various regions across the parietal, frontal, and temporal cortex that reduce neural activity while performing complex cognition tasks [26]. The PIGD subgroup is related to faster disease progression with increasing dementia risk [27]. A previous study depicted the significantly reduced functional connectivity between the left DLSFG and left SMA, suggesting that the cognition networks are involved in the gait problem [28]. The discovery of an increased connection between the CB and the DMN networks in PIGD‐PD patients is a noteworthy advancement in the field of neuroscience. This change in connectivity implies that there is a more intricate brain relationship in patients who are having difficulty walking [29]. During the fMRI dual‐task, heightened cognitive activity and diminished motor activity were observed in the cerebellum. The changes in the structure of the cerebellum were shown to be associated with higher levels of brain activity in the cognitive regions of the cerebellum, as well as poorer performance in executive and attentive tasks [30]. Studies have highlighted that cerebellar atrophy in PD has implications for network connectivity. The presence of negative differences between PD and controls suggests a decrease in functional cerebello‐cortical connectivity due to cerebellar atrophy. On the other hand, positive differences indicate an increase in connectivity as a result of cerebellar atrophy, possibly indicating a compensatory mechanism [31]. When the brain is at rest, the stronger link between CB and CEN regions may be a way for the brain to make up for the fact that cerebellar motor areas and the basal ganglia aren't working as well [32].

Patients with PIGD‐PD spend more time in state 1 and less time in state 4, which is consistent with previous research [10]. The occurrence of brain function sparse connectivity state 1 shown increased significantly in PD patients, accompanied by an extended duration, suggesting that PD patients spend considerable periods of time in a condition of diminished functional connection, particularly in PIGD‐PD patients. Connectivity state 1 with poor connectivity may suggest significant impairment of the brain's network. The phenomenon of DFNC has been observed in several neuropsychiatric disorders, such as depression, Alzheimer's disease, and dementia with Lewy bodies [33, 34, 35]. These alterations persist for longer durations when there are fewer connections across brain regions. Gottlich et al. categorized PD as a “disconnection syndrome” marked by heightened connectivity and reduced interaction among brain modules [36]. This aligns with data suggesting that effective communication within a network is crucial for carrying out physical movements [37]. We established a correlation between the severity of MDT at stage 1 and the presence of PIGD symptoms. An increased amount of time spent in state 1 was linked to a worsening gait severity. PIGD‐PD patients have an increased duration in weakly connected DFNC states, suggesting a decrease in inter‐network communication [38]. Furthermore, there was a negative correlation between symptoms of PIGD and NT, perhaps leading to impaired motor cortex functioning in terms of accurate and timely information processing [39].

## 5 Conclusion

Our research focused on examining the alterations in both static and dynamic connections in individuals with PIGD‐PD. Our findings indicate that the decline in connectivity between CB and SMN leads to the deterioration of the SMN in these patients. Patients with PIGD‐PD were shown to have a greater possibility of being held in a state of limited neural connections and an inability to translate information. In this situation, there was an increase in the link between the cerebellum and DMN networks. This increase is believed to be a form of dynamic compensation.

## Author Contributions

Design and conceptualization: Li Zhang and Yuncheng Wu. Data statistics and analysis: Yixuan Zhang, Qun Yao, Feng Han, Shengrong Li, Qi Zhu, and Daoqiang Zhang. Acquisition of data: Yinyin Jiang, Yaxi Wang, Xu Jiang, Yang Zhao, Haiying Zhang, Shuangshuang Dong, Dongfeng Li, Yaning Chen, Yang Pan, and Jun Yan. Writing of the manuscript: Bo Shen. Supervision: Li Zhang and Yuncheng Wu. All authors read and approved the final paper.

## Conflicts of Interest

The authors declare no conflicts of interest.

## Data Availability

The data that support the findings of this study are available on request from the corresponding author. The data are not publicly available due to privacy or ethical restrictions.
